# Peripheral ulcerative keratitis secondary to tuberculosis: A case report and literature review

**DOI:** 10.1097/MD.0000000000039482

**Published:** 2024-08-30

**Authors:** Shuang Wang, Yajie Gong, Keke Huang, Jun Huang

**Affiliations:** a Ophthalmology Department, Chengdu Third People’s Hospital, Southwest Jiaotong University, Chengdu, Sichuan, China; b Department of Microbiology, Chengdu Third People’s Hospital, Southwest Jiaotong University, Chengdu, Sichuan, China.

**Keywords:** peripheral ulcerative keratitis, pulmonary tuberculosis, secondary glaucoma, tuberculosis, uveitis

## Abstract

**Rationale:**

Compared with intraocular tuberculosis, ocular tuberculosis with ocular surface involvement is rare. Corneal involvement in ocular tuberculosis may include interstitial keratitis or peripheral ulcerative keratitis. We report a case of peripheral ulcerative keratitis directly caused by tuberculosis.

**Patient concerns:**

A 20-year-old man complained of vision loss and pain in the left eye that had lasted for 1 week. A slit lamp examination of the left eye showed a corneal epithelial defect, interstitial corneal edema, and a white irregular infiltrative lesion and ulcer (with the dimension of 2 × 2.5 mm) in the inferior temporal region.

**Diagnoses:**

The corneal ulcer was scraped, and the *Mycobacterium tuberculosis* deoxyribonucleic acid polymerase chain reaction was positive.

**Interventions and outcomes:**

After a month of oral antituberculosis treatment, the corneal ulcer resolved, and the intraocular inflammation improved.

**Lessons:**

Peripheral ulcerative keratitis secondary to tuberculosis can be directly caused by *M tuberculosis*.

## 1. Introduction

Tuberculosis is a common public health problem,^[1]^ especially in the Tibet region in China. It most commonly affects the lungs, and ocular tuberculosis is relatively rare. Ocular tuberculosis accounts for 6.8% of all tuberculosis cases.^[2]^ While it can affect all eye tissues,^[3]^ corneal tuberculosis is extremely rare.^[4]^ Tuberculosis may cause interstitial keratitis or peripheral ulcerative keratitis (PUK), which is characterized by progressive peripheral corneal stromal thinning and an associated epithelial defect.^[5]^ Ocular lesions can be caused by a direct invasion of microorganisms or result from immunologic reactions (delayed hypersensitivity type IV) in the absence of the infectious agent.^[4]^ We report a case of peripheral ulcerative keratitis caused by direct invasion of *Mycobacterium tuberculosis*.

## 2. Case presentation

**Figure 1.**
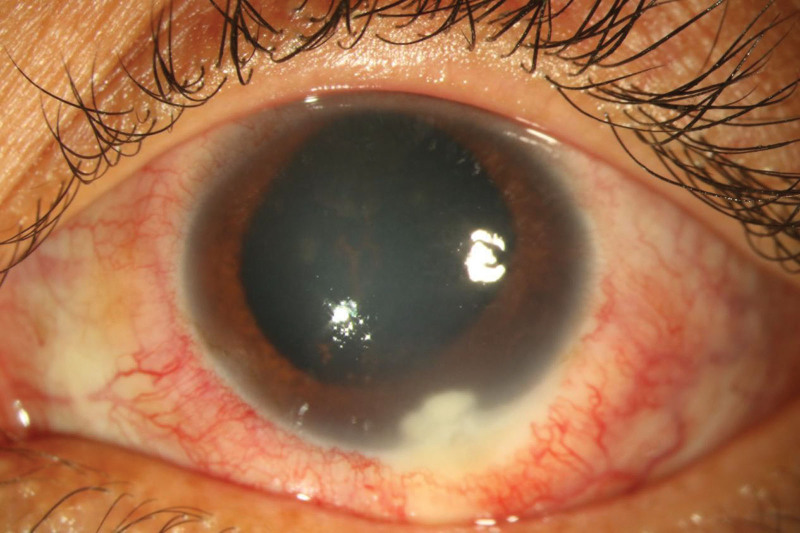
Edema of the corneal stroma and a 2 × 2-mm white irregular infiltrating lesion and ulcer below the temporal area. Keratic precipitates (+) and flare (++) are observed in the anterior chamber. The lens is transparent, and the fundus cannot be viewed. The best-corrected visual acuity was counting fingers for the left eye and 20/20 (Snellen chart) for the right eye, and the intraocular pressure was 18 mm Hg for the right eye and 65 mm Hg for the left eye.

A 20-year-old man complained of vision loss and pain in the left eye that had lasted for 1 week. Slit lamp examination of the left eye showed a peripheral corneal epithelial defect, interstitial corneal edema, and a white irregular infiltrative lesion and ulcer (with a dimension of 2 × 2.5 mm) in the inferotemporal region (Fig. 1). The anterior chamber findings included several keratic precipitates, flare (++), and pigmented exudation. The fundus could not be viewed because of the corneal edema. The best-corrected visual acuity was counting fingers for the left eye and 20/20 (Snellen chart) for the right eye. The intraocular pressures were 18 and 65 mm Hg for the right and left eyes, respectively. PUK, secondary glaucoma, and uveitis were diagnosed. The patient received intravenous mannitol (250 mL, a single dose), 2% cartenolol, 0.2% brimonidine, tobramycin, and dexamethasone eye drops.

The patient, a native of Linzhi County in Tibet Province and a year-round resident was from an area in China with a high incidence of tuberculosis. Clinical symptoms, such as weight loss, malaise, and fever, were not mentioned by the patient. He had no other medical or lifestyle history such as keeping pets. Systemic examinations, including the purified protein derivative skin test (induration of 10 mm), were carried out. The enzyme-linked immunospot assay for interferon-γ was strongly positive, with an elevated erythrocyte sedimentation rate. The other blood tests (such as antinuclear antibody, rheumatoid factor, antineutrophil cytoplasmic antibody, and human immunodeficiency virus) were negative. Chest computed tomography showed multiple spots and nodules in the lung. We highly suspected ocular tuberculosis-related lesions and the peripheral ulcerative lesions were scraped and sent for microbiological examination. Acid-fast staining was negative, while *M. tuberculosis* deoxyribonucleic acid polymerase chain reaction was positive. Pulmonary fibrobronchoscopy, sputum acid-fast staining, and *M. tuberculosis* deoxyribonucleic acid polymerase chain reaction were negative. Ocular and pulmonary tuberculosis were diagnosed. Oral antituberculosis treatment was initiated with isoniazid, rifampicin, pyrazinamide, and ethambutol based on the previous examination.

**Figure 2.**
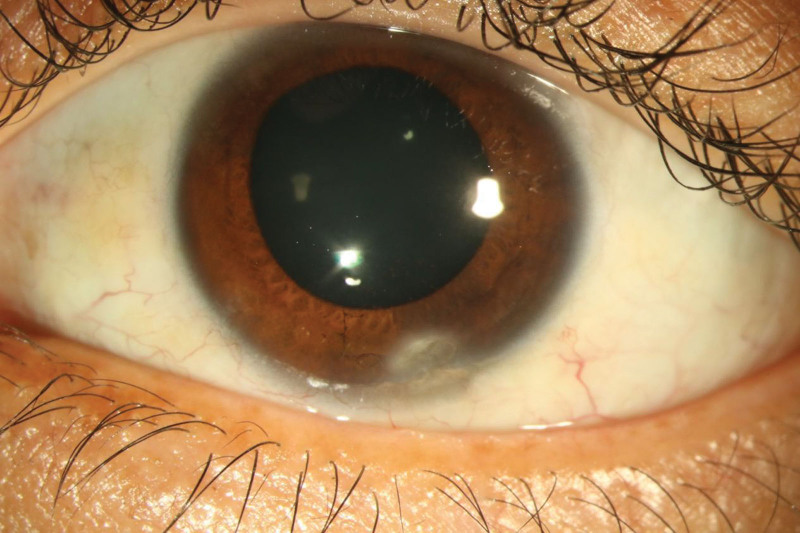
The corneal ulcer was completely controlled after a month of oral antituberculosis treatment. The anterior chamber was quiet, and the conjunctival congestion had resolved. There were no cells in the anterior chamber.

The corneal edema resolved, the anterior chamber flare disappeared (−), best-corrected visual acuity improved to 2/20, and intraocular pressure was 20 mm Hg after one week of topical treatment. The slit lamp examination of the left eye revealed cells in the vitreous cavity. Fundus examination revealed a flattened retina with scattered circular yellow and white lesions close to the blood vessels on the retina. Optical coherence tomography showed a normal macula. The corneal ulcer resolved after a month of oral antituberculosis treatment (Fig. 2), and the circular yellow and white lesions on the retina had disappeared. The topical treatment with eye drops was discontinued, and the 4-drug oral antituberculosis therapy was continued for another month, followed by a 2-month continuation treatment with isoniazid and rifampicin. The patient was lost to follow-up afterward.

## 3. Discussion

Tuberculosis can lead to interstitial keratitis or PUK, with the latter being relatively rarer. We conducted a literature review (Table 1) to illustrate the involvement of the cornea in tuberculosis.^[4,6–13]^ We found that direct corneal invasion by *M. tuberculosis* was extremely rare, especially for PUK.

**Table 1:** A literature review about the involvement of the cornea in tuberculosis.

Author	Age	Sex	Clinical symptoms	Origin	Direct/indirect	Treatment
Rafiezadeh et al^[5]^	84	Male	Autoimmune keratitis	Lung	Indirect	Topical steroids
Yangzes et al^[6]^	Middle age	Male	Cornea interstitial keratitis with corneal perforation	Lung	Indirect	Oral antitubercular therapy and oral steroids
Kamal et al^[7]^	17	Female	Bilateral interstitial keratitis and granulomatous uveitis	No primary site was detected	Indirect	Oral antitubercular therapy and topical steroids
Arora et al^[8]^	11	Female	Bilateral disciform keratitis	Spine	Indirect	Oral antitubercular therapy and topical steroids
Costa et al^[9]^	10	Not available	Cornea interstitial keratitis and sclero-uveitis	Brain	Indirect	Oral antitubercular therapy and topical steroids
Serban et al^[10]^	34	Female	Interstitial keratitis	Right wrist joint	Indirect	Oral antitubercular therapy and topical steroids
Arora et al^[11]^	16	Female	Peripheral ulcerative keratitis	Lung	Indirect	Oral antitubercular therapy and topical steroids
Anitha et al^[12]^	28	Male	Bilateral peripheral ulcerative keratitis	Lung	Indirect	Oral antitubercular therapy and topical steroids
Gupta et al^[13]^	47	Male	Peripheral ulcerative keratitis and necrotizing scleritis	No primary site was detected	Direct	Oral antitubercular therapy and topical steroids

In this case, PUK was caused by the direct invasion of *M. tuberculosis* rather than an immune response indicated based on the corneal ulcer scraping results. Despite negative results from multiple sputum examinations, the presence of lung lesions on computed tomography scans suggested pulmonary infection by *M. tuberculosis*. We hypothesized that pulmonary infection precedes corneal lesions, with *M. tuberculosis* potentially spreading from the lungs to the corneoscleral limbus via the bloodstream, thereby inducing the corneal ulcer. The ulcer located at the edge of the corneoscleral rather than the center of the cornea suggested that the pathogen was more likely to originate from the bloodstream rather than direct external infection. The diagnosis of this patient was based on findings from the corneal scraping and microbiological examination. It is extremely rare to obtain *M. tuberculosis* directly from the corneal tissue.

In addition to corneal involvement, there was active inflammation in the anterior and posterior segments of the eye. We did not perform pathogen testing on intraocular fluid (anterior chamber fluid and vitreous fluid), and we did not know if the inflammation was caused by direct infection by the pathogens or immune reactions to the pathogens. However, we speculated that the anterior chamber inflammation was caused by an immune response triggered by the ulcer, while the retinal inflammation was directly caused by pathogens because retinal nodules were close to the blood vessels. The patient had a peripheral corneal ulcer, but it did not lead to corneal perforation. The intraocular inflammation of the patient improved significantly with a marked reduction in the corneal edema after the topical corticosteroid treatment and before the oral antituberculosis drug treatment. However, the ulcer did not heal. We believed that the intraocular condition was more likely caused by an immune response rather than direct infection by *M. tuberculosis*. The corneal ulcer, due to the direct invasion of *M. tuberculosis*, was cured after the treatment with oral antituberculosis drugs, which was completely different from the results obtained with the topical corticosteroid use.

## 4. Conclusion

We have reported a case of PUK secondary to tuberculosis caused by direct invasion by *M. tuberculosis. M. tuberculosis* deoxyribonucleic acid polymerase chain reaction using the corneal scraping yielded a positive result. The corneal ulcer resolved and the intraocular inflammation improved after a month of oral antituberculosis treatment. Regular antituberculosis treatment is important and necessary irrespective of whether inflammation is caused by pathogens or an immune response.

## Author contributions

**Writing – original draft:** Shuang Wang, Yajie Gong.

**Writing – review and editing:** Shuang Wang, Yajie Gong, keke Huang.

**Methodology:** Jun Huang.
